# Exercise and Omentin: Their Role in the Crosstalk Between Muscle and Adipose Tissues in Type 2 Diabetes *Mellitus* Rat Models

**DOI:** 10.3389/fphys.2018.01881

**Published:** 2019-01-07

**Authors:** Cynthia Aparecida de Castro, Karina Ana da Silva, Marina Campos Rocha, Marcela Sene-Fiorese, Keico Okino Nonaka, Iran Malavazi, Fernanda de Freitas Anibal, Ana Cláudia Garcia de Oliveira Duarte

**Affiliations:** ^1^Department of Physiological Sciences, Federal University of São Carlos, São Paulo, Brazil; ^2^Department of Genetic and Evolution, Federal University of São Carlos, São Paulo, Brazil; ^3^Department of Morphology and Pathology, Federal University of São Carlos, São Paulo, Brazil; ^4^Department of Physical Education, Federal University of São Carlos, São Paulo, Brazil

**Keywords:** adipokines, cytokines, diabetes, exercise, inflammatory

## Abstract

This study aims to analyze the effects of resisted, aerobic, and combined exercises on omentin levels in visceral adipose tissue and muscle of rats with experimental diabetes to verify whether these adipokines are related to the glucose pathway and inflammation process in this model. Male Wistar rats received a high-fat diet for 4 weeks and a low dose of streptozotocin (35 mg/kg) to induce experimental diabetes. After inducing diabetes, the animals were divided into 4 experimental groups (*n* = 10): diabetic control (C); resistance training (RT); aerobic training (AT); and combined training (CT). The groups were exercised for 12 weeks, 3 times/week, where: RT means the stair climbing protocol until exhaustion; AT is the 30 min/day reaching 20 m/min protocol, and CT is the combination of RT and AT. The AT group showed reduced retroperitoneal and mesenteric adipose tissue and abdominal fat deposits. Our study also showed a possible control of blood glucose, as well as decreased Interleukin 6 (IL-6) and C-reactive protein, increased circulating adiponectin and increased omentin in visceral adipose tissue. In addition, the AT group affected the glucose pathway by stimulating phosphorylation of Akt in muscle tissue. Omentin also showed a strong positive correlation with adiponectin and a moderate negative correlation with IL-6. Thus, our findings indicated that omentin in type 2 diabetes is changed by AT. Furthermore, increased omentin levels had a close association with the glucose pathway by stimulating phosphorylation of Akt in muscle tissue and with IL-6 in serum, suggesting that omentin is likely to have anti-inflammatory and protective action in experimental diabetes.

## Introduction

For decades, it was believed that adipose tissue was only a reservoir of fat. However, today it is clear that adipose tissue is also an endocrine organ that secretes and releases peptides with hormonal functions into the circulation (Cinti, 2005; Duarte et al., 2008). Thus, adipose tissue produces many adipokines, which play an important role in modulating energy homeostasis and insulin sensitivity.

Omentin-1, also known as intelectin-1, is an adipokine identified in adipose tissue that is also related to obesity and its comorbidities. This adipokine is preferably expressed in visceral adipose tissue (visceral vascular stroma cells), which is negatively associated with insulin resistance and obesity (Herder et al., 2015), increasing the effect of insulin on the glucose metabolism (Yang et al., 2006). This increases glucose uptake and transport by insulin stimulated Akt activation (serine/threonine kinase Akt), possibly acting as one of the proteins involved in insulin signaling.

Previous studies reported that obesity ( Yang et al., 2006; Shibata et al., 2012) and type 2 diabetes (Tan et al., 2010) are associated with decreased omentin levels, however aerobic training (AT) (Saremi et al., 2010; Wilms et al., 2015) increases the concentration of omentin-1. Therefore, discovering new alternatives to prevent the accumulation of visceral fat and mobilization of adipokines is of the utmost importance when modulating energy homeostasis and insulin sensitivity. Studies have shown that aerobic or anaerobic exercises may be effective (Zanuso et al., 2010) in the treatment of obesity (Figueiredo et al., 2017) and diabetes (Plomgaard and Weigert, 2017). Exercise promotes increases in the systemic levels of anti-inflammatory cytokines such as Interleukin-4 (IL-4), Interleukin (IL-10) (Balducci et al., 2010) and adiponectin (Miyazaki et al., 2010), and stimulates the C-X-C motif chemokine ligand 8 (CXCL8), a cytokine that acts as an angiogenic factor in human microvascular endothelial cells (Ostrowski et al., 2001), and is associated with insulin resistance and obesity (Bruun et al., 2003). Exercise induced a decrease in the pro-inflammatory cytokines, such as tumor necrosis factor α (TNF-α) and IL-6, C-reactive protein (CRP), thereby assisting the prevention or mitigation of such chronic-degenerative diseases such as obesity and diabetes (Kasapis and Thompson, 2005; Petersen and Pedersen, 2005).

In addition, physical exercise has been considered an important tool in the treatment of obesity and type 2 diabetes, acting on metabolic inflexibility (Sene-Fiorese et al., 2015). Studies have considered the hypothesis that dysfunction in peripheral tissues, such as skeletal muscle and adipose tissue, represent the etiology of the development of metabolic inflexibility (Saltiel and Kahn, 2001) due to the inability of increasing glucose oxidation during insulin stimulation (Galgani et al., 2008; Jung and Choi, 2014). Considering the complexity of the physiological mechanisms of insulin resistance in diabetes and obesity related to metabolic flexibility in different tissues, crosstalk between muscle and adipose tissues needs to be established as both are essential components in this process (Saltiel and Kahn, 2001).

However, the relationships between omentin-1 with the metabolic changes are currently associated with diabetes and the effect of exercise in these cases is still unclear. Thus, this study aims to examine the effects of resistance, aerobic, and combined exercises on omentin-1 in visceral adipose tissue and muscle of rats with an experimental diabetes model to verify if this adipokine is related to the glycemic pathway and the inflammatory profile.

## Materials and Methods

### Experimental Animals

All procedures were approved by the Ethics Committee at the Federal University of São Carlos (Protocol number 008/2013). For this study, 40 male Wistar rats (age – 45 days) were acclimated in individual cages under controlled temperature, humidity, and lighting (12-h dark/light cycle) with free access to water and food.

### Diabetes Induction

For diabetes induction, the rats were fed palatable hyperlipidic diets (Estadella et al., 2004) for 4 weeks and received an intraperitoneal dose of Streptozotocin (Sigma^®^) 35 mg/kg body weight (pH 4.4 streptozotocin in citrate solution), without previous fasting (Castro et al., 2017). Diabetic animals were considered those with glycemic levels ≥250 mg/dl (Srinivasan et al., 2005). The remaining animals were randomly divided into 4 groups with 10 rats each: Diabetic Sedentary (CS); Diabetic resistance training (RT); Diabetic AT; and Diabetic combined training (CT). The animals were fed daily and their body weight was measured once a week.

### Exercise-Training Protocols

The training procedures were carried out after inducing diabetes lasting 12 weeks, with 2 weeks adaptation, and 10 weeks of progression. All sessions were held for 3 days per week.

### Aerobic Training

Aerobic training consisted of treadmill running. The animals underwent a protocol with programed time and progressive speed for three days per week, reaching a duration of 30 min at a speed of 20 m/min at the end of the experiment. During the first 2 weeks, the animals were subjected to an adaptation process. This training program lasted 10 consecutive weeks (Irigoyen et al., 2005).

### Resistance Training

Initially, rats were familiarized with strength training, which consisted of a stair climbing system (1.1 m × 0.18 m; 2 cm spacing between grid steps, 80th grade) with a loading apparatus fixed in their tails. At the top of the ladder, there was a cage (20 cm × 20 cm × 20 cm) in which the animals were able to rest for 120 s. This procedure was repeated until the rats voluntarily went up the stairs three times consecutively, without encouraging the clamped tail. After 2 weeks of adaptation, a maximum load test was carried out with the experimental groups, and starting progressive resistance exercise. The first training session consisted of climbing the ladder four to eight times, gradually carrying heavier loads. In the initial climb, a load of 75% of the animal body weight was applied. Once this stage was completed, an additional weight of 30 g was added to the apparatus. This procedure was repeated successively until the final load reached a weight whereby the rats could no longer climb. This final load was considered the “maximum load” for the rats for that particular session. During the first four climbing movements, the rats carried 50, 75, 90, and 100% of the maximum load and in subsequent climbing, an additional load of 30 g was added until the rats obtained a new maximum loading capacity (Hornberger and Farrar, 2004).

### Combined Training

The combined-training protocol consisted of the sum of the two protocols: RT during the morning and AT in the afternoon, 3 days per week, with a minimum interval of 6-h inter sessions. The adaptation and progression periods were similar to the other training procedures. After 72 h of the last training session, the rats were euthanized by decapitation and the serum was separated. Visceral adipose tissue and gastrocnemius muscle were dissected, weighed, immediately frozen in liquid nitrogen, and stored at -80 C for subsequent analyses.

### Glucose and Insulin Tolerance Tests (ITT)

Blood glucose concentrations were measured weekly by the tail (a drop of blood was obtained after a small incision was made at the tip of the tail), using an Accu-Check^®^glucose meter (Roche Diagnostic, Indianapolis, United States). An ITT was performed after inducing diabetes (6 weeks), as well as after 12 weeks of training. After overnight fasting, anesthetized rats were intraperitoneally injected with human insulin 1 U/kg BW. Blood samples were obtained from the tail vein before injection, and also after the insulin injection (0, 15, 30, and 45 min after) (Ropelle et al., 2006).

### Biochemical Analyses

Serum was obtained by the centrifugation of blood samples, and the aliquots were used to measure triacylglycerol, cholesterol, and high-density lipoprotein (HDL) cholesterol. For these measurements, we used Labtest Diagnostic S.A. commercials kits (Minas Gerais, Brazil).

### Determination of Immunoenzymatic Assay Method (ELISA)

The quantification of plasma cytokines, adiponectin, insulin, and omentin were performed using the immunoenzymatic assay method (ELISA). For this purpose, the collected blood from each animal (5 ml) was placed into tubes without an anticoagulant for approximately 2 h until it coagulated. Then, the samples were centrifuged at 3500 rpm for 15 min. The serum resulting from this centrifugation was aliquoted into microtubes and frozen at -80°C. Commercial kits were used for this analysis and Interleukin 4 (IL-4), interleukin 6 (IL-6), tumor necrosis factor alpha (TNF-α), and Interleukin 10 (IL-10) were measured using ELISA kits from OptEIA (BD Biosciences, Pharmingen, San Diego, United States). C-X-C motif chemokine ligand 8 (CXCL8), C-reactive protein (CRP), and Adiponectin were evaluated using Duo Set kits (R&D Systems, Minnesota, United States). The omentin was obtained with Rat EIA-OME (RayBiotech^®^, Norcross, GA, United States). Quantitative measurements of insulin were taken using the Abnova kit (Abnova Corporation, Taipei, Taiwan). All the used kits followed the manufacturer’s recommendations. The results were expressed in pg/ml or ng/ml.

### Western Blotting

The quantification of omentin was evaluated by western blotting assays. Visceral fat and muscle were homogenized in a lysis buffer [25 mM Tris-HCl -pH 7.4, 0.5 mM EDTA, 0.5 mM EGTA, 0.05% Triton X-100, 10 mM β-mercaptoethanol, and mini-complete protease inhibitors (Roche Diagnostics, Indianapolis, IN, United States)] and centrifuged for 30 min at 4°C, 14,000 g in a microcentrifuge. Protein concentrations of the supernatants were determined according to Lowry et al. (1951). Samples containing 30 mg of protein extracts were fractionated by SDS-PAGE electrophoresis (12% gel), transferred to nitrocellulose (GE) membranes, and the system was submerged in a transfer system (BioRad). The membranes were incubated with specific primary antibodies (Anti-Omentina 1:1000, sc-104334, Santa Cruz^®^; Anti-AMPK 1:1000, #2793, Anti-Phosfo-AMPK (Thr172) 1:100. #2535, Anti- Akt 1:1000, #4691, Anti-Phosfo-Akt (Ser473) 1:250, #9271, and Cell Signaling^®^) and the transfer process took place overnight at 4^o^C. The membranes were washed thoroughly with TBS/0.1% Tween-20 at 4 sessions of 5 min before incubation with the secondary antibodies (Anti-mouseIgG-HRP 1:3000, #7076, Cell Signaling^®^; Anti-goat IgG-HRP 1:3000, sc-2020, and Santa Cruz^®^) for 2 h at room temperature. Protein concentrations were normalized by using β-actin diluted 1:2,000 (Cell Signaling Technology, Beverly, MA, United States) in the visceral fat, or GAPDH diluted 1:10,000 (Abcam) in muscle. β-actin was detected with mouse peroxidase (HRP) – conjugated with a second antibody (Cell Signaling) diluted 1:3,000 in TBS-T, and GAPDH using antirabbit antibody (Cell Signaling), incubated and agitated for 1 h at room temperature. The antibody complexes were visualized using chemiluminescence (ECL reagents, GE). The band intensity was determined using the Versa Doc 5000 Imaging system (Bio-Rad Laboratories, Hercules, CA, United States).

### Statistical Analysis

Statistical analysis used the unpaired and paired Student’s *t*-tests for the baseline groups. Comparisons among the groups were made using the parametric one-way ANOVA; where *F* ratios were significant, and further comparisons were made using the *Bonferroni* test. The results were expressed as the means ± SD accompanied by the indicated number of rats used in the experiments. *P* < 0.05 was considered statistically significant. The relation between serum cytokines and adipokines in the adipose tissue and in muscle was evaluated by linear regression and Pearson’s correlation analysis.

## Results

### General Characteristics of the Rats

**FIGURE 1:**
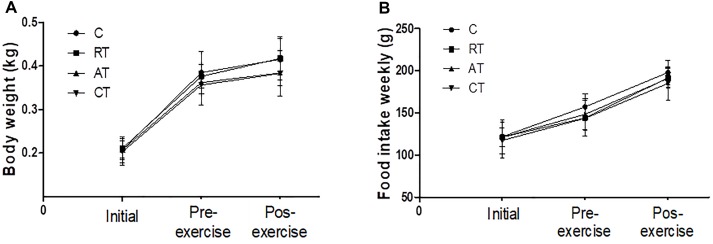
Characteristics of the experimental groups. **(A)** Body weight and **(B)** Food intake assessments at the beginning of the experiment (Initial), after inducing diabetes and before the exercise protocol (Pre-exercise) and after 12 weeks of exercise (Post-exercise). C, control group; RT, resistance training; AT, aerobic training; CT, combined training. Data are expressed as means ± SD.

The body weight (Figure 1A) and food intake values (Figure 1B) were similar in all the experimental groups during the study period. There was an expected progressive increase in weight and consumption until diabetes was induced, but when exercise occurred, we observed that the weight stabilized, even though the caloric consumption increased.

### Effect of Exercise on Adipose Tissue Depots

In order to understand the relationship between exercise and visceral adipose tissue weights, we investigated changes at the sizes of three types of adipose tissues: mesenteric adipose tissue (MES), retroperitoneal adipose tissue (RET), and epididymal adipose tissue (EPI). This study demonstrated that the AT reduced fat in the MES and EPI, as the CT reduces only in RET (Table 1).

**Table 1:** Mass of adipose tissue depots.

	Adipose tissue depots (g)		
	EPI	RET	MES
C	1.80 ± 0.44	1.57 ± 0.78	0.89 ± 0.27
RT	1.46 ± 0.40	1.28 ± 0.43	0.77 ± 0.23
AT	1.25 ± 0.39^∗^	0.96 ± 0.31	0.59 ± 0.14^∗^
CT	1.31 ± 0.34	0.89 ± 0.22^∗^	0.61 ± 0.16


*Results expressed in means ± SD (n = 10 animals per group). C, control; RT, resistance training; AT, aerobic training; CT, combined training; MES, mesenteric adipose tissue; RET, retroperitoneal adipose tissue; and EPI, epididymal adipose tissue. P < 0.05. ^∗^compared to C.*

### Effect of Exercise on Glucose and Insulin Sensitivity

**FIGURE 2:**
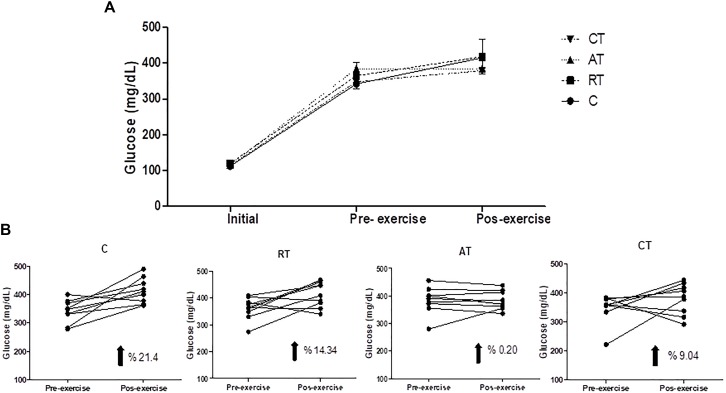
Progression of glucose during the experiment. **(A)** Glucose in all experimental groups. **(B)** Evolution of glucose per group. Assessments at three times: beginning of the experiment (Initial); after the diabetes induction and before the exercise protocol (Pre-exercise) and after 12 weeks of exercise (Post-exercise). C, control group; RT, resistance training; AT, aerobic training; CT, combined training. Data are expressed as means ± SD.

As shown in Figure 2A, the rats became progressively hyperglycemic during the study period and reached the fasting plasma glucose concentration of C on the day of the initial exercise. In the final exercise, the blood glucose concentration in rats was only marginally elevated during the exercise protocol, but the AT prevented the increase of glucose. The C group had a 21.4% increase in glycemia, whereas the RT group had a 14.34% increase. The combined CT group had 9.04% and the AT group had less than 1% (Figure 2B).

**FIGURE 3:**
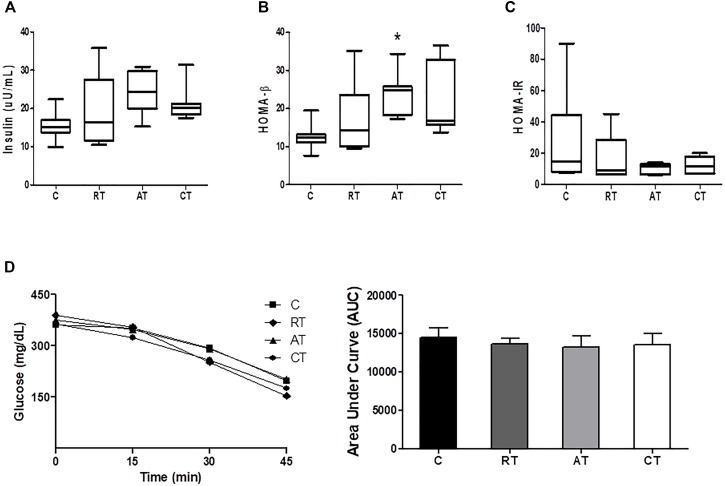
Insulin sensitivity assessments. **(A)** Insulin. **(B)** HOMA-β: homeostatic model assessment of cell β function. **(C)** HOMA-IR: homeostatic model assessment of insulin resistance. **(D)** Insulin Tolerance Test (ITT), with the glycemic curve of each group and area under curve (AUC). Data are expressed as means ± SD. *P* < 0.05. ^∗^, compared to C.

Insulin concentration was not altered by training (Figure 3A), which was also observed in the intraperitoneal ITT (Figure 3D) and in the HOMA-IR, despite a reduction in the exercised groups (Figure 3C). On the other hand, in HOMA-β, which estimates the function of β-cells, insulin concentration was higher in the AT group (Figure 3B).

### Effect of Exercise on Inflammation

**FIGURE 4:**
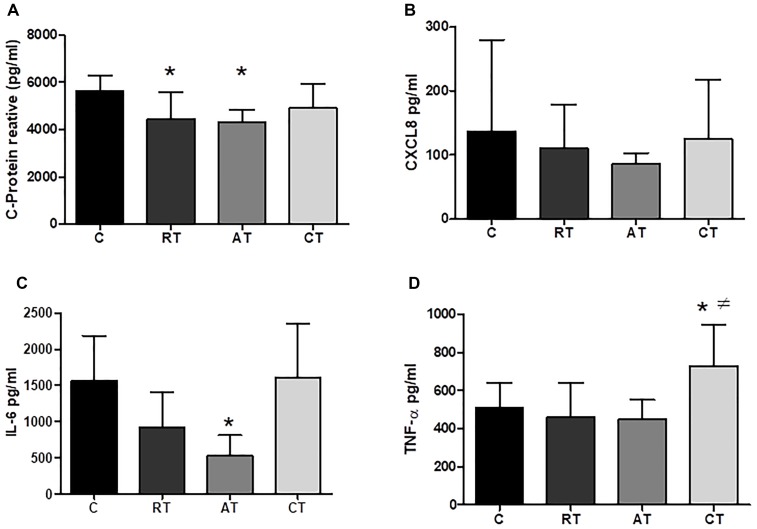
Pro-inflammatory cytokines and CPR in serum. **(A)** C-protein reactive. **(B)** CXCL8 (C-X-C motif chemokine ligand 8). **(C)** Interleukin 6. **(D)** Tumor necrosis factor alfa. C, control group; RT, resistance training; AT, aerobic training; CT, combined training. Data are expressed as means ± SD. *P* < 0.05. ^∗^, compared to C; #, compared to AT.

**FIGURE 5:**
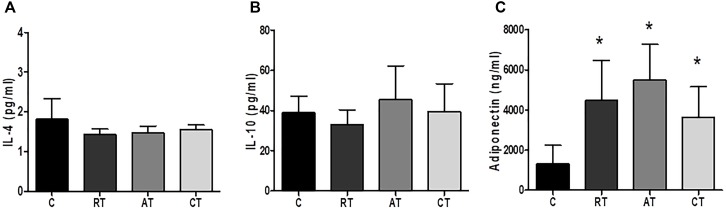
Anti-inflammatory cytokines and adiponectin in serum. **(A)** Interleukin 4. **(B)** Interleukin 10. **(C)** Adiponectin. C, control group; RT, resistance training; AT, aerobic training; CT, combined training. Data are expressed as means ± SD. *P* < 0.05. ^∗^, compared to C.

The effects of long-term exercise on serum concentration of inflammatory markers were examined. Figure 4A shows that C-reactive protein (CRP) was reduced when the three exercise protocols were carried out, but only AT and CT presented a statistical difference compared to C. The IL-6 levels were significantly reduced by AT when compared to C (Figure 4C). The TNF-α levels were not altered in the RT and AT groups, whereas, the CT group increased the TNF-α significantly when compared to the C group (Figure 4D). The levels of CXCL8 chemokine (C-X-C motif chemokine ligand 8) showed no significant difference between the groups (Figure 4B). Considering the anti-inflammatory markers, IL-4 and IL-10 were not altered by the exercise protocols (Figures 5A,B). The adiponectin was significantly higher in the three studied groups: RT, AT, and CT compared to C (Figure 5C).

### Effect of Exercise on Omentin in Serum and Adipose Tissue

**FIGURE 6:**
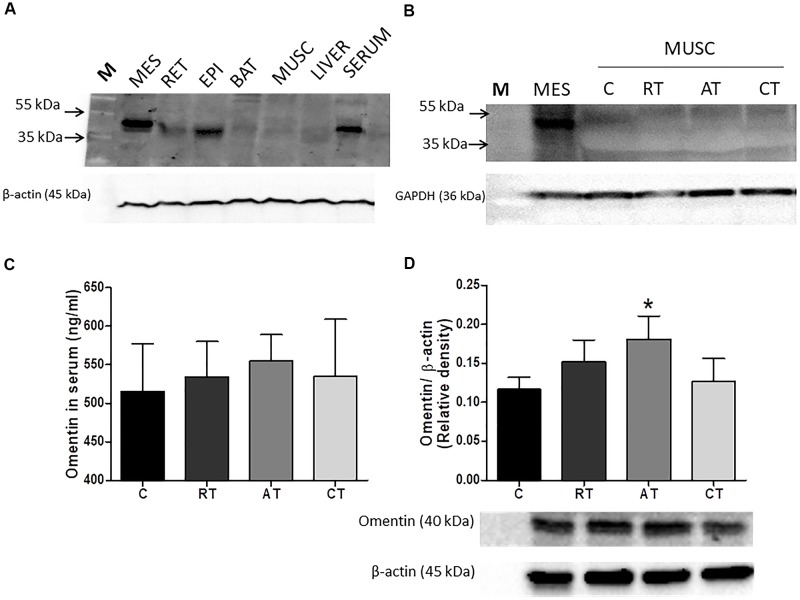
Western blot representative of omentin in diabetic animals. **(A)** in tissues: Molecular Weight Marker (M); Mesenteric Adipose Tissue (MES), Retroperitoneal (RET), Epididimal (EPI), Brown Adipose Tissue (BAT), Liver, and Serum. For this result, 3 independent replicates were performed. **(B)** Representative membrane with 4 sample of omentin in muscle (one per group) compared to MES, showed that omentin was not found in muscle (I = 6 sample per group). **(C)** Serum Omentin Values (ELISA) (*n* = 10 sample per group). **(D)** Omentin (40 kDa) in MES in the experimental protocols. C, control group; RT, resistance training; AT, aerobic training; CT, combined training. Results are presented as the relative density after normalizing with β-actin protein. Data are expressed as means ± SD (*n* = 6 sample per group). *P* < 0.05. ^∗^, compared to C.

Multiple tissue western blot analysis (Figure 6A) indicated that omentin is highly expressed in MES and is also present in serum, but less expressed in EPI and RET. This cytokine is virtually undetectable in muscle, liver and brown adipose tissue (Figure 6A). Further analysis confirmed no detection of omentin in the muscle (Figure 6B) when compared to the visceral adipose tissue sample as a control. The concentration of omentin-1 in the visceral adipose tissue (Figure 6D) was higher only in the TAD when compared to the diabetic sedentary group (C) control. However, the systemic concentration of omentin was not altered through training interventions (Figure 6C).

### Effect of Exercise on Akt and AMPK Phosphorylation in Muscle

**FIGURE 7:**
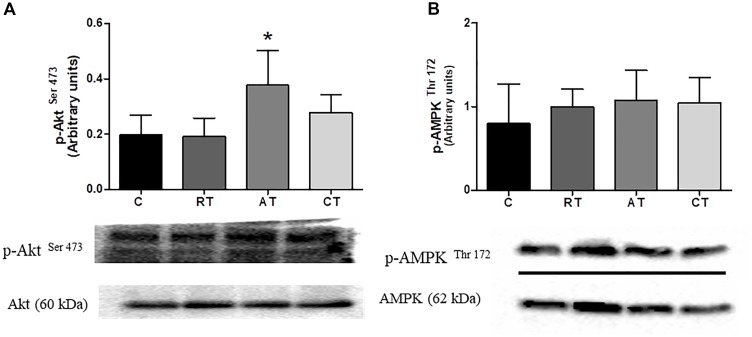
Effect of exercise on Akt and AMPK phosphorylation in muscle. **(A)** Akt phosphorylation. **(B)** AMPK phosphorylation C, control group. RT, resistance training; AT, aerobic training; CT, combined training. Results are presented as relative density after normalizing the with Total Akt or Total AMPK protein. Data are expressed as means ± SD (*n* = 6 sample per group), *P* < 0.05. ^∗^, compared to C.

In the muscle, the phosphorylated Akt was quantified by the western blot method. The phosphorylated Akt relative to total Akt was greater only in the AT group when compared to group C (Figure 7A), and the phosphorylated AMPK relative to the total AMPK was not altered by the exercise protocols (Figure 7B).

### Correlations of Omentin and Inflammation

**FIGURE 8:**
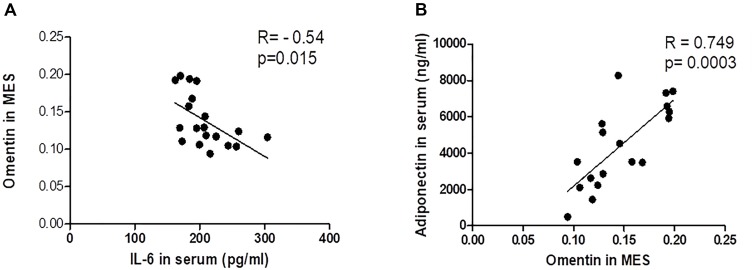
Correlation between Omentin in Visceral Adipose Tissue with Systemic IL-6 and Adiponectin. **(A)** Omentin x IL-6. **(B)** Omentin x Adiponectin.

Our data demonstrate the correlation between serum omentin, IL-6 and adiponectin. The adiponectin appears to modulate visceral omentin-1 expression and may be negatively correlated with serum IL-6 (*r* = -0.54, *P* = 0.015 moderate negative correlation) (Figure 8A). On the other hand, the expression of visceral omentin is positively correlated with circulating adiponectin concentration (*r* = 0.749, *P* = 0.0003, strong positive correlation) (Figure 8B).

## Discussion

The present study describes that the AT was more efficient in reducing abdominal fat deposits than the resistance and CT protocols studied. Furthermore, AT prevented increased blood glucose, decreased Interleukin 6 (IL-6), and C-reactive protein; increased circulating adiponectin and increased omentin in visceral adipose tissue. In addition, it affected the glucose pathway by stimulating phosphorylation of Akt in muscle tissue.

Therefore, the studies strongly suggest the importance of non-pharmacological treatment for obesity and diabetes, such as exercise, and this has been widely studied (Alizadeh et al., 2017; AminiLari et al., 2017; García-Hermoso et al., 2017). Thus, the effects of exercise on the physiology of adipose tissue may vary according to the type and amount of exercise. Previous studies have shown that RT and swimming positively affected the parameters of inflammation, body weight, and adipocyte areas in obese rats (Speretta et al., 2012). On the other hand, the intermittent exercise was more efficient than the continuous one in reducing the effects of a high fat diet and sedentary lifestyle by inducing an improvement in visceral and central adiposity (Sene-Fiorese et al., 2008). These are important features that prove the efficacy of exercise, which depends on parameters such as intensity, frequency and duration, and promotes different metabolic adaptations in adipose tissue (Thompson et al., 2012). All these responses may be involved in the release of adipokines that will act in an autocrine and paracrine way in the individual (Kusminski et al., 2016).

As previously mentioned, body mass regulation was recognized as an important mechanism in the crosstalk between muscular and adipose tissue, and is essential for the maintenance of homeostasis (Moreno-Navarrete et al., 2013). Thus, recent studies have demonstrated a great importance of adipokines in the development and progression of disorders related to obesity and metabolic syndrome, as well as in the pathogenesis of type 2 diabetes (Stanford et al., 2015; Faramarzi et al., 2016). The interest in these adipokines comes from the attempt to find a non-invasive way to control these diseases. Recently, it has been reported that the omentin is produced and secreted by visceral adipose tissue when compared to the subcutaneous tissue in obese humans (Yang et al., 2006). Indeed, omentin in diabetic animals is expressed differently in each fat deposit and serum. Omentin was highly expressed in MES, and slightly less expressed in epididymal adipose tissue (EPI) and retroperitoneal (RET) tissue; undetectable in muscle, liver, and brown adipose tissue; and is also high in serum. The action of omentin in these organs is not well known as there is no conclusion about its receptors and its mechanism of action. Thus, it is believed that it can exert a function in several organs, including muscle, liver and adipose tissue, increasing insulin sensitivity, and altering glucose metabolism (Auguet et al., 2011).

In our study, we observed that training increased omentin protein expression in the MES, and among the three exercise protocols studied, the AT promoted greater expression in this tissue. Studies have shown that the expression of omentin in adipose tissue is negatively correlated with the obesity index, insulin resistance, and parameters of lipid metabolism, so that its decrease may contribute to the aggravation of diseases correlated with insulin resistance (Cai et al., 2009). Alizadeh et al. (2017) showed that high intensity interval exercise in obese rats and experimental type 1 diabetes caused an increase in gene expression of omentin in adipose tissue. In fact, AT increased omentin in the MES and improved β-cell function, which indicates an improvement in insulin sensitivity and consequently an improvement in glycemia, even in untreated diabetic rats without their diet being altered (even caloric consumption). It is worth noting that the animals continued to be fed on a hyperlipidemic diet until the end of the experiment. Derosa et al. (2013) showed decreased insulin resistance with increased β- cell function in the obese/diabetic groups, after omentin application. Thus, this is the first study to show the behavior of omentin in adipose tissue and the three exercise protocols (Resistance, Aerobic, and Combined) in the experimental type 2 diabetes model.

Regarding the levels of circulating omentin, there is no consensus in the literature regarding its relation to exercise. Madsen et al. (2015) demonstrated that strenuous exercise and moderate exercise, performed 8 times per week, promoted increased serum omentin in ovariectomized rats, which are also a model of obesity (Babaei et al., 2015). On the other hand, a study on acute aerobic exercise (75% VO_2_max) showed a decrease in serum omentin levels in obese rats when compared with eutrophic exercises (Huang et al., 2016), which demonstrates the difference of modulation before the metabolic alterations of each disease. Other studies found no change in circulating omentin levels after 3 months of AT (3 times/week for 30 min), despite a significant reduction in body weight, fasting insulin, and HOMA-IR (Urbanová et al., 2014; Faramarzi et al., 2016). The hyperlipidic diet may have contributed to worsening beneficial results promoted by physical exercise, as the animals consumed the hyperlipidic diet until the end of the experiment.

In subcutaneous adipose tissue and in visceral adipocytes, omentin increases glucose transport and the phosphorylation of Akt, stimulated by insulin (Yang et al., 2006), which suggests that omentin may improve the sensitivity to this hormone. Thus, the need to investigate a possible action of omentin on the muscle tissue of trained diabetic rats is clear. However, our study did not detect the presence of omentin in the muscle of these animals. Nevertheless, our study brought a possible action of omentin on the muscle of diabetic rats. However, further analysis is needed to investigate whether these exercise protocols would be able to alter the phosphorylation of Akt and AMPK as these two proteins are involved in the signaling of the GLUT-4 transporter (Petersen and Shulman, 2002).

Insulin stimulates Akt phosphorylation and its activity, whereas muscle contraction activates AMPK through phosphorylation of the Thr^172^ site and allosteric activation by AMP. Muscle contraction may also activate Akt, but the importance of this signaling pathway for glucose uptake is still unclear (Vendelbo et al., 2014). Thus, intracellular signaling for the transport of glucose in muscle is stimulated by different pathways, either through insulin or physical exercise. In our study, we found that the AT group did not significantly present higher phosphorylation of AMPK, even though it had an elevation of 35%, but there was a greater phosphorylation of Akt in the muscle. These results suggest that even in conditions of decompensated diabetes, induced exercise can cause beneficial changes in the insulin-signaling pathway by increasing omentin concentration, as observed in adipose tissue. However, more studies are necessary to understand the performance of omentin in this organ as there are no conclusions about its receptors and its mechanism of action in the tissues in general. Thus, it is believed that it may exert a function in several organs, increasing insulin sensitivity, and altering glucose metabolism (Auguet et al., 2011) acting via membrane receptor activation.

In the present study, resistance and AT were able to decrease CRP and IL-6 and there was an improvement in the anti-inflammatory system by stimulating higher circulating levels of adiponectin, without significantly depressing the immune system since there was no reduction in circulating anti-inflammatory cytokines, such as IL-4 and IL-10.

Another important effect of exercise is the reduction of inflammatory response due to the reduction of adipose tissue. However, the results are still conflicting, since some studies did not observe changes in serum concentrations of adiponectin (Ahmadizad et al., 2007; Praet and Van Loon, 2009). On the other hand, the results found in this study are consistent with the notion that exercise increases adiponectin concentrations, and may therefore mediate the effects of insulin resistance, glycaemia, and lipidemia (Blüher et al., 2006; Passos and Gonçalves, 2014). Regarding adiponectin, studies have reported an increase in their serum concentrations in response to aerobic and resisted exercises (Blüher et al., 2006; Balducci et al., 2010). These results agree with the assumption that there may be a dose-response relation of intensity in RT and an increase in adiponectin (Simpson and Singh, 2008; Hopps et al., 2011). In addition, it is worth mentioning that we observed a direct relationship between increases in omentin and adiponectin levels, which was demonstrated by the strong positive correlation in our study (*r* = 0.749, *P* = 0.0003), corroborated in the literature (Yan et al., 2011; Feng et al., 2013). This correlation is important because it helps us clarify the role of omentin, since adiponectin is an adipocytokine whose increase in plasma levels is related to decreased insulin resistance, improved insulin sensitivity, and increased anti-inflammatory markers (Li et al., 2013). Accordingly, the literature indicates that omentin regulation may be dependent on adiponectin (Yan et al., 2011), however, this is not clear considering the current available data. Other authors consider that adiponectin and omentin, especially when synthesized from subcutaneous adipose tissue, might be the most important adipokines in the regulating obesity, insulin sensitivity and T2DM (Sitticharoon et al., 2014).

Although combined exercise had a greater effect on the reduction of retroperitoneal intra-abdominal adipose tissue, the animals had higher levels of the inflammatory markers: TNF-α and IL-6. These results may be related to the fact that these markers increase in response to very strenuous exercises due to muscle damage in acute exercise (Pedersen et al., 2007) considering that even 54 h after exercise, the inflammatory levels still remained high. However, the role of IL-6 in metabolism is controversial as its increased plasma levels are associated with T2DM. Moreover, studies show that these increases may be involved with muscle myogenesis and increased fat oxidation (Benatti and Pedersen, 2015). In this case, acute elevations in IL-6 may inhibit the production of TNF-α by decreasing inflammation. In this study, we did not confirm IL-6 performance as anti-inflammatory as TNF levels increased with combined exercise, triggering an inflammatory response (Karstoft and Pedersen, 2016). Another important fact is that IL-6 increases the production of IL-10 (Pedersen and Febbraio, 2008), helping the anti-inflammatory action, which was also not observed in this study where there was no enucleation of the plasma IL-10 cytokine. Thus, low-grade chronic inflammation has been shown as the key feature in the pathogenesis of insulin resistance and type 2 diabetes *mellitus* (T2DM) (Hotamisligil, 2006; Ouchi et al., 2011). The role of adipose tissue in this context is very significant as it acts as an endocrine organ, secreting anti and pro-inflammatory hormones and cytokines, as well as numerous adipokines that play a central role in inflammation (Piya et al., 2013). This causes the endogenous production of pro-inflammatory cytokines by adipocytes, which increases and maintains the inflammatory response in tissues such as liver, muscle, and adipose tissue, stimulating the transcription of the pro-inflammatory molecules IL-1β, TNF-α, IL-6, C-reactive protein, and chemokines (MCP-1, CXCL8) (Hernández-Díaz et al., 2015).

Training has been known to cause adaptations in white adipose tissue, including reductions in cell size, adipokine production, and modulation of inflammation (Duarte et al., 2008; Miyazaki et al., 2010; Hopps et al., 2011). In this context, the main findings of our study were that the different types of training were able to alter omentin expression in the visceral adipose tissue of diabetic rats. These effects are associated with biochemical changes and improved insulin sensitivity. In addition, we confirmed that omentin expression in adipose tissue is negatively correlated with circulating IL-6 and positively correlated with adiponectin in serum.

Thus, the results of this study confirm that exercise is a tool capable of modulating visceral adipose tissue, increasing the production of Omentin, which could prevent the progression of experimental type 2 diabetes in these animals. Although the cause-and-effect relationship is still unclear, the effects of exercise may be due to modulation of omentin. These data may result in improvements in blood glucose levels in untreated diabetic animals, even if these changes are only at the autocrine level as this increase in tissue growth did not significantly reflect their circulating levels. Therefore, further studies are needed to determine the specific performance of omentin against the etiology of obesity and type 2 diabetes. Our results suggest that there is a crosstalk of skeletal muscle and adipose tissue relative to omentin-1 so much so that it is related to glucose metabolism and mainly to insulin sensitivity. In our study, the hyperlipidic diet was offered continuously for diabetic animals which could explain the different circulating omentin levels observed from the results in the literature. It is known that the results of training are potentiated with the consumption of food and calorie control, especially in type 2 diabetes cases. Thus, our model suggests that exercise protocols can contribute to important changes in the metabolic condition and the prevention of diabetes.

## Author Contributions

CC, main author of the work, participated in all the techniques and analyses of this work, made the graphs, tables, figures, and helped to write the article. KS actively participated in the techniques and developed and carried out the experimental model. MR participated in the molecular analyses with Western Blot and Real Time in her laboratory and contributed to the analysis and discussion of the results of this step. MS-F actively participated in the biochemical techniques and participated in the discussion of the results in this step. KN participated in the analysis, discussion and formulation of the results, as well as writing the article. IM contributed to availability of the necessary equipment for techniques to prepare the Western Blot and Real Time in his laboratory and participated in the analysis and discussion of the results of this step. FA contributed to availability of the necessary equipment for techniques to prepare the immunoenzymatic assay (ELISA), participated in the analysis, discussion, and formulation of the results, and writing the article. AD, main advisor of the project, actively participated in the work and in the techniques developed and participated in the analysis, discussion, and formulation of the results.

## Conflict of Interest Statement

The authors declare that the research was conducted in the absence of any commercial or financial relationships that could be construed as a potential conflict of interest.
